# The Electrical and Photodetector Characteristics of the Graphene:PVA/p-Si Schottky Structures Depending on Illumination Intensities

**DOI:** 10.1021/acsomega.4c05219

**Published:** 2024-07-11

**Authors:** Murat Ulusoy, Serhat Koçyiğit, Adem Tataroğlu, S. Altındal Yerişkin

**Affiliations:** † Department of Physics, Faculty of Science, Gazi University, Teknikokullar, 06500 Ankara, Türkiye; ‡ Central Laboratory Application and Research Centre, Bingol University, 12000 Bingol, Türkiye; § Department of Chemistry and Chemical Processing Technologies, Vocational Highschool of Technical Sciences, Gazi University, Teknikokullar, 06500 Ankara, Türkiye

## Abstract

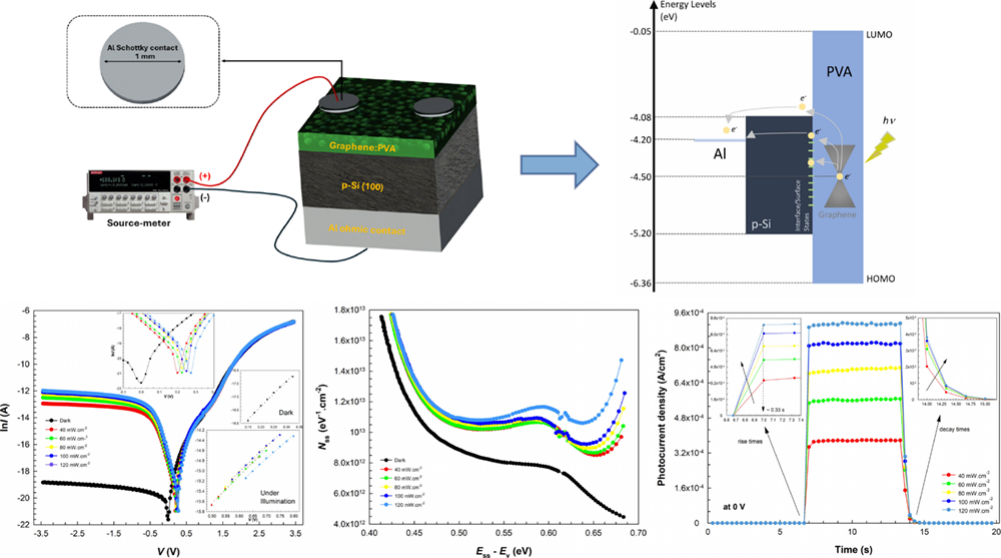

Five samples were fabricated to obtain a diode with a PVA interface, both with and without graphene doping at different rates with high rectification in the dark. The electrospinning method was employed to apply the doped and undoped solutions, creating the interlayers. Since the diode with a 1 wt % graphene-doped PVA interlayer outperformed the other samples, the main electrical and photodetector characteristics of this structure were investigated. The electrical parameters of the diode were probed by the TE, Norde, and Cheung methods, and the parameters (*n* and ϕ_B_) acquired by both approaches were significantly influenced by illumination and voltages. The interface/surface state intensity values (*N*_ss_) were also calculated in the dark and under each illumination as a function of the band/energy gap depth (*E*_ss_–*E*_v_). The time-dependent steady-state conditions and rise-decay behavior of the photocurrents during illumination were also investigated. Due to the high photocurrent values, the photosensitivity at zero bias is approximately 1.4 × 10^4^ at 100 mW cm^–2^. The responsivity and detectivity values appear to be altered significantly with changes in the illumination and voltage. Additionally, a double logarithmic plot of *I*_ph_ vs *P* reveals good linearity with slope values ranging from 0.5 to 1.

## 1 Introduction

The photovoltaic (PV) effect typically results in the generation of an electric current in a semiconductor device upon exposure to light. Depending on its energy, light absorbed on a diode surface can cause electron–hole (e^*–*^–h^*+*^) pairs to form at the junction. The e^*–*^–h^*+*^ pairs separate under the internal electric field of the depletion layer, generating a photocurrent in the diode, and thus, higher-order currents are observed for more pairs generated.^1,2^ These photogenerated charge carriers depend on the illumination/light intensity absorbed by the surface. Due to this scenario, a current is generated in addition to the dark current, especially in the reverse region. In other words, the reverse bias current changes in proportion to the illumination intensity, and this additional current is called photocurrent.^3,4^ The interlayers of PV cells play a crucial role in determining their optoelectronic properties. Factors such as the organic/inorganic nature, composition, thickness, and shapes of these interlayers significantly impact the performance of the devices. Incorporating an organic-based active layer or photoactive medium at the interlayer provides various benefits. Since sensitive organic light detectors can be fabricated for photodiode applications on almost any substrate or surface, whether flat or curved, they are highly favored in advanced optoelectronic devices. These detectors, known as organic photodiodes (OPDs), operate based on the distinctive electrical properties of the organic interlayer material.^5,6^ OPDs are ever increasingly used in imaging technologies and photosensing/detecting applications, as they provide the optimal balance between quick response, linearity, and processability.^7^ In addition to these, they have attractive features such as easy and cheap production processes, lightweight, and reasonable surface spreading rate.^8^

For the purpose of photoelectric characterization, this study utilized poly(vinyl alcohol) (PVA) as the preferred interlayer. Employing organic polymers in line with various purposes, including PVA, is quite common in producing OPDs.^8−11^ Due to its superior chemical and physical properties, PVA is a water-soluble polymer widely preferred by researchers. Some distinguishing properties are optical transmission, thin film formation, flexibility, crystallization due to the H bonds between PVA chains, ability to adhesive, noncorrosive nature, and easy solubility.^12^ In addition, the chemical effects of certain nanostructures dispersed in the PVA matrix can modify the optical, chemical, and physical properties of the polymer.^13−16^ Many types of nanofillers have been used for this modification. However, graphene and/or graphene oxide, in particular, can be brought to the forefront with its superior properties, further improving the nature of PVA itself.^17^

Similarly, it has been observed that acquiring the large surface area of graphene or graphene-containing composite structures into PVA increases the possibility of charge carrier generation and, thus, photoelectric performance.^10,18^ The superior mechanical and electrical properties of graphene obtained experimentally at the beginning of the 21st century are indisputably the focus of intense interest in many researchers today. Its high thermal and electrical conductivity, unique elasticity, high fracture strength, and extensive surface area per weight are clearly some of the main reasons it is frequently used in many electronic or optoelectronic devices, either as a monolayer^19^ or in combination with different compounds/structures.^20−24^ The exceptional photosensing capabilities of graphene are a crucial subject in scientific research due to their ability to produce hot electrons, leading to photocurrents in response to light. This field of study continues to be momentous. In particular, graphene, which possesses weak electron–phonon coupling, stands out as an ideal active interfacial medium with its ability to facilitate carrier multiplication in very short times (fs).^25^

Because of the superior properties of graphene-doped PVA compared to undoped PVA, the main goal of the present study is to produce a diode with an organic polymer interlayer and to investigate its usability in PV or optical sensor applications. For this aim, in the first step, a reference specimen with only PVA at its interlayer and specimens with different weight proportions of the graphene-doped PVA composite interlayer were prepared, and their *I*-*V* characteristics were examined in the dark. In the measurements executed, it was observed that the structure with the lowest leakage current, the highest rectification ratio (RR), and the optimal linear region was the structure with a 1 wt % graphene-doped PVA interlayer. Based on this, the illumination-dependent measurements of this structure were executed in the ±3.5 V range with 25 mV steps and 40–120 mW cm^–2^ range with 20 mW cm^–2^ steps. The structure’s RR values were determined to be 1.67 × 10^5^ and 1.67 × 10^2^ under dark and 120 mW cm^–2^ conditions, respectively. The study also investigated the time-dependent steady-state conditions and rise-decay behavior of photocurrents during illumination. Steady-state conditions of the photocurrents were generally stable over time under illumination and at certain voltage levels. Some photodiode characteristics, such as sensitivity, responsivity, and detectivity, were obtained as a strong function of the voltage and illumination intensities. Finally, we point out that this study examines the rectification properties of n-type Si-based diodes with varying graphene doping ratios. The results obtained are used to discuss the detector characteristics of the structure in a factual, unambiguous manner, thus differing from prior studies.^11,26,27^ Moreover, photodetector parameters of similar heterostructures are presented for comparison just before the Conclusion section in Table 4.

## 2 Experimental Section

A p-type silicon substrate with a (100) orientation, 1–10 Ω·cm resistivity, and a thickness of 350 μm was employed to create the specimens. Detailed experimental information can be found in the referenced work,^28^ as all processes, such as all chemical and physical cleaning steps, the creation of various interlayers, and the fabrication of contacts, are exactly similar to our previous study. Poly(vinyl alcohol) (PVA) (*M*_w_ 85,000–124,000 g/mol) obtained from Sigma-Aldrich was used as the polymeric precursor in the coating applied to the Si wafer layer subsequent to the ohmic contact process via the electrospinning method. Graphene powder had a 5–8 nm diameter and was obtained from Grafen Chem. Ind. Ethanol (96% v/v) was preferred as the solvent for graphene dispersion and was obtained from TEKKİM company. Graphene was first mixed in ethanol at a ratio of 1:10 and dispersed with an ultrasonic shaker at room temperature for 24 h to prepare the coated polymer solution. Simultaneously, to prepare the aqueous solution of PVA (10%), PVA powder was first added to distilled water and mixed for 2 h at a constant temperature of 80 °C. The prepared solution was kept at room temperature for approximately 24 h to eliminate the bubbles. The mixtures were added to each other to contain 1% graphene compared to the PVA content of the solution. Thus, the solution was made ready for the electro-spin coating process. In order to coat with the electro-spin method, three contrivances are required. These are known as DC high voltage power supply (Gamma High Elec. Res. Inc., ES30P-20W/DAM), syringe pump (New Era Syringe Pump), and metal collector (aluminum plate). During the coating phase of the electrospinning method, noncoated p-Si wafers were clipped onto the metal collector, and the solution to be coated was drawn into the syringe. Thanks to the electric field between the metal tip of the syringe and the metal collector, the solutions in the syringe were coated on the p-Si wafers on the metal collector, forming nanofiber structures. A JEOL JSM 6510 brand SEM-EDX device and a RIGAKU ULTIMA IV brand XRD device were used in the characterization stages.

**Figure 1:**
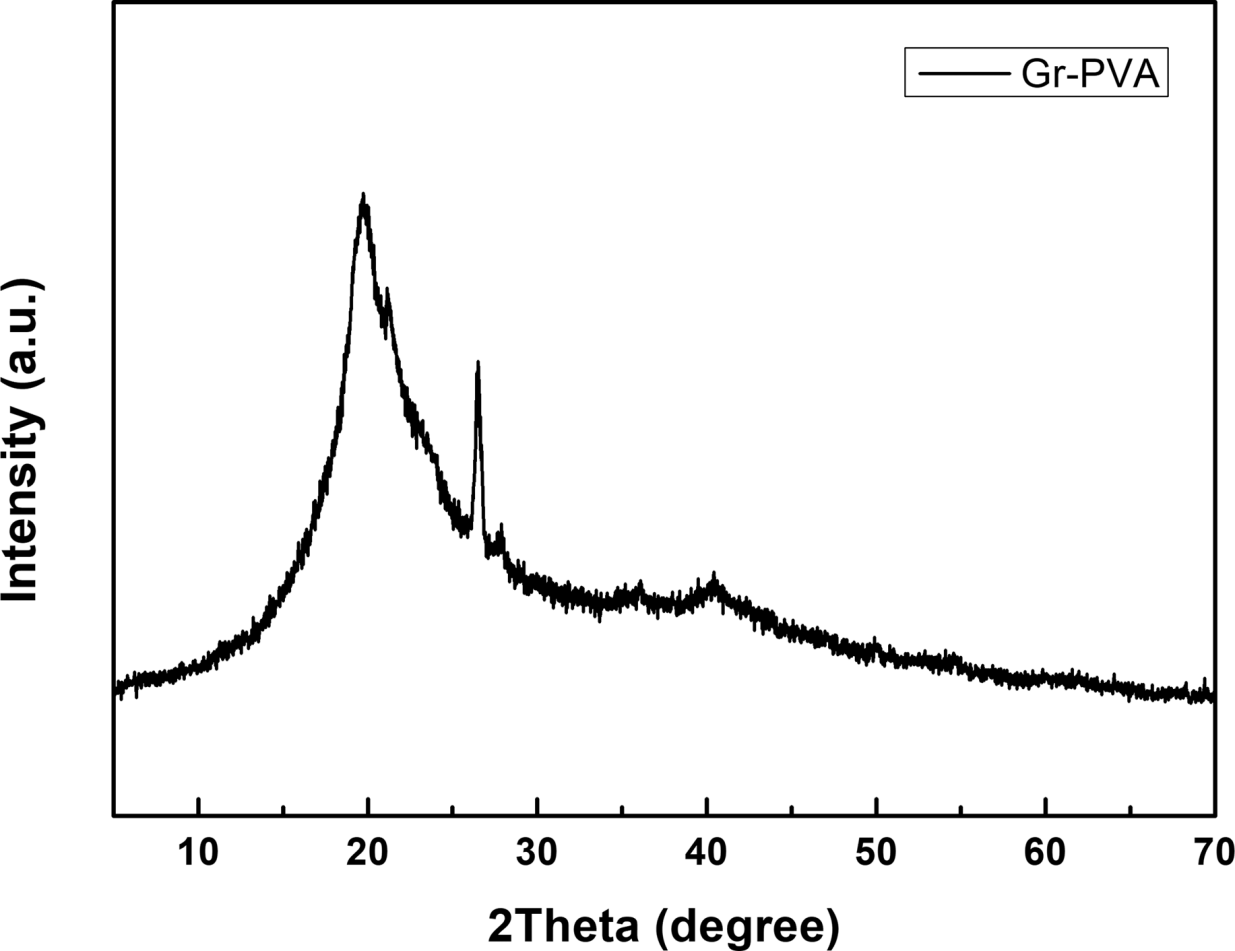
XRD pattern of Gr-doped PVA.

The XRD pattern of the PVA-graphene electrospun coating on the ohmic contact is given in Figure 1. A broad and long peak with 2theta values between 14° and 30°, a long peak at 2θ = 26.46°, a broad and small peak with 2theta values between 39° and 42° were observed according to this pattern. It was determined from literature data that the 2theta values between the peaks as 14° and 30° and between the peaks as 39° and 42° originated from PVA.^29^ The peak at 26.46° was also compatible with graphite-2H, which corresponds to PDF Card No.: 00-041-1487. Because normally, in the literature data, the intensity of the PVA peak at 14°–30° was equal to the peak intensities of the starting point (14°) and the end point (30°), in this sample, it was seen that the peak intensity of the starting point is lower than that of the end point. It was understood from literature data that this difference was due to graphene.^29^

**Figure 2:**
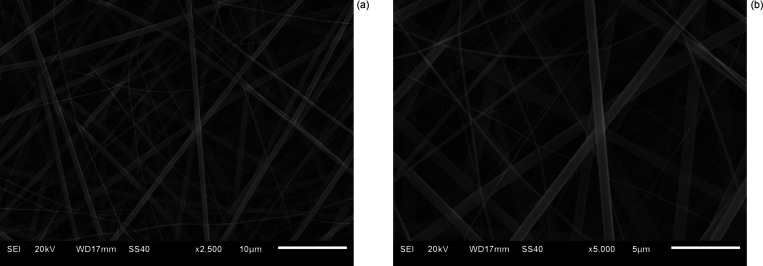
SEM images of PVA-graphene electrospun coating (a) at 2500× magnification and (b) at 5000× magnification.

SEM images of the PVA-graphene electrospun coating are given in Figure 2. In these images, images were taken of the electrospun coating at 2500× magnification in Figure 2a and at 5000× magnification in Figure 2b. When these images were examined, it was determined that the electrospinned sample formed nanofibers, and the electrospinning process was successful. The bending structures are formed in nanofibers in some places, but generally, the formation of straight fiber structures was observed from the images. Thin and thick nanofiber structures were determined, and the calculations showed that the diameters of thin nanofibers varied between 73 and 174 nm and thick ones varied between 571 and 996 nm. Graphene was added to the PVA polymer, and no other active ingredient was included. For this reason, with graphene doping, homogenization changed. The structures with different diameters, thin diameter, and thick diameter were formed due to the dispersion effect outside the normal distribution.

EDX analysis of PVA-graphene electrospun coating is given in Table 1, and the analysis was measured from a wide image at 100× magnification. C, O, and Si elements were detected in the sample. Since no other element has been found besides these elements, there was no impurity in the sample. It was thought that the C element originates from PVA and graphene, the O element from PVA, and the Si element from p-Si wafers.

**Table 1:** EDX Analysis of PVA-Graphene Electrospun Coating

element	line	intensity (c/s)	error 2-sig	conc	units
C	Ka	349.70	4.965	38.779	wt %
O	Ka	493.19	5.824	26.923	wt %
Si	Ka	5,235.05	18.842	34.299	wt %
			**total**	**100.000**	**wt %**

**Figure 3:**
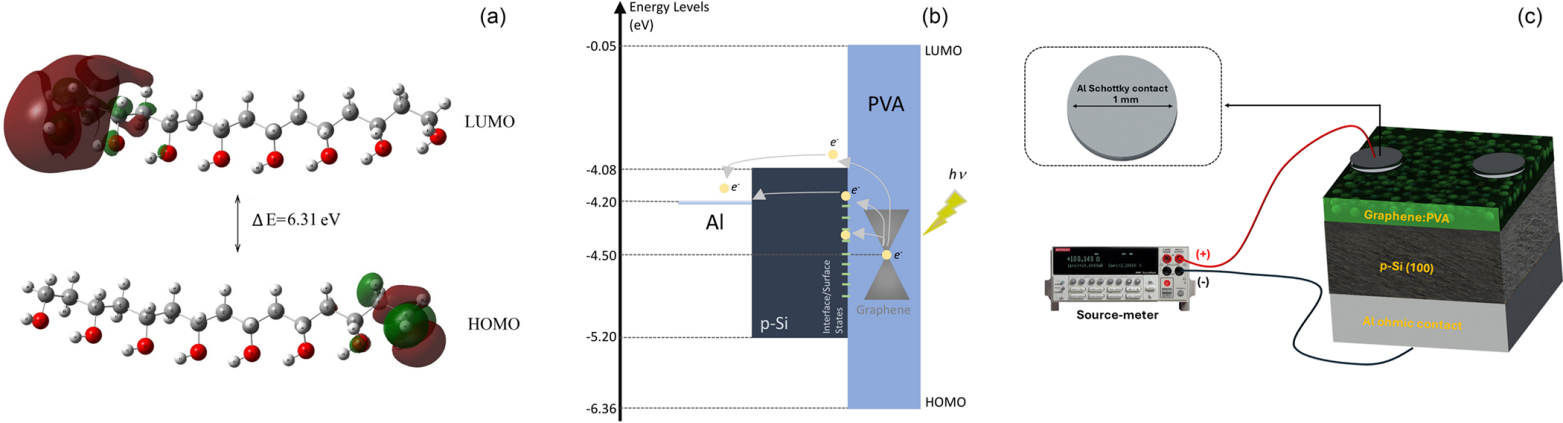
(a) HOMO and LUMO contour maps of PVA molecule. (b) Energy band diagrams of the structures. (c) Visual of the measurement system and preferred diode.

Furthermore, the highest occupied molecular orbital (HOMO) and lowest unoccupied molecular orbital (LUMO) energies were calculated for PVA. The HOMO energy corresponds to the ionization potential, while the LUMO energy corresponds to electron affinity.^30^ The calculation of the energy gap between HOMO and LUMO is crucial for detecting molecular electrical transport. The 3D approximate structure of PVA was drawn with the GaussView package program. Optimizations and calculations of HOMO–LUMO energies were carried out with the Gaussian09 program. The LUMO level holds a calculated energy of −0.05 eV, while the HOMO level holds a calculated energy of −6.36 eV. This results in an energy gap of 6.31 eV. Additionally, Figure 3a depicts the HOMO and LUMO contour maps of PVA in a vacuum medium. The negative parts of the molecule are represented in red, and the positive parts are in green.

A Newport–Oriel light source was harnessed to illuminate the surface. A schematic representation of the preferred diode and energy band diagrams for the structures is depicted in Figure 3b,c.

## 3 Results and Discussion

### 3.1 Electrical Characteristics

**Figure 4:**
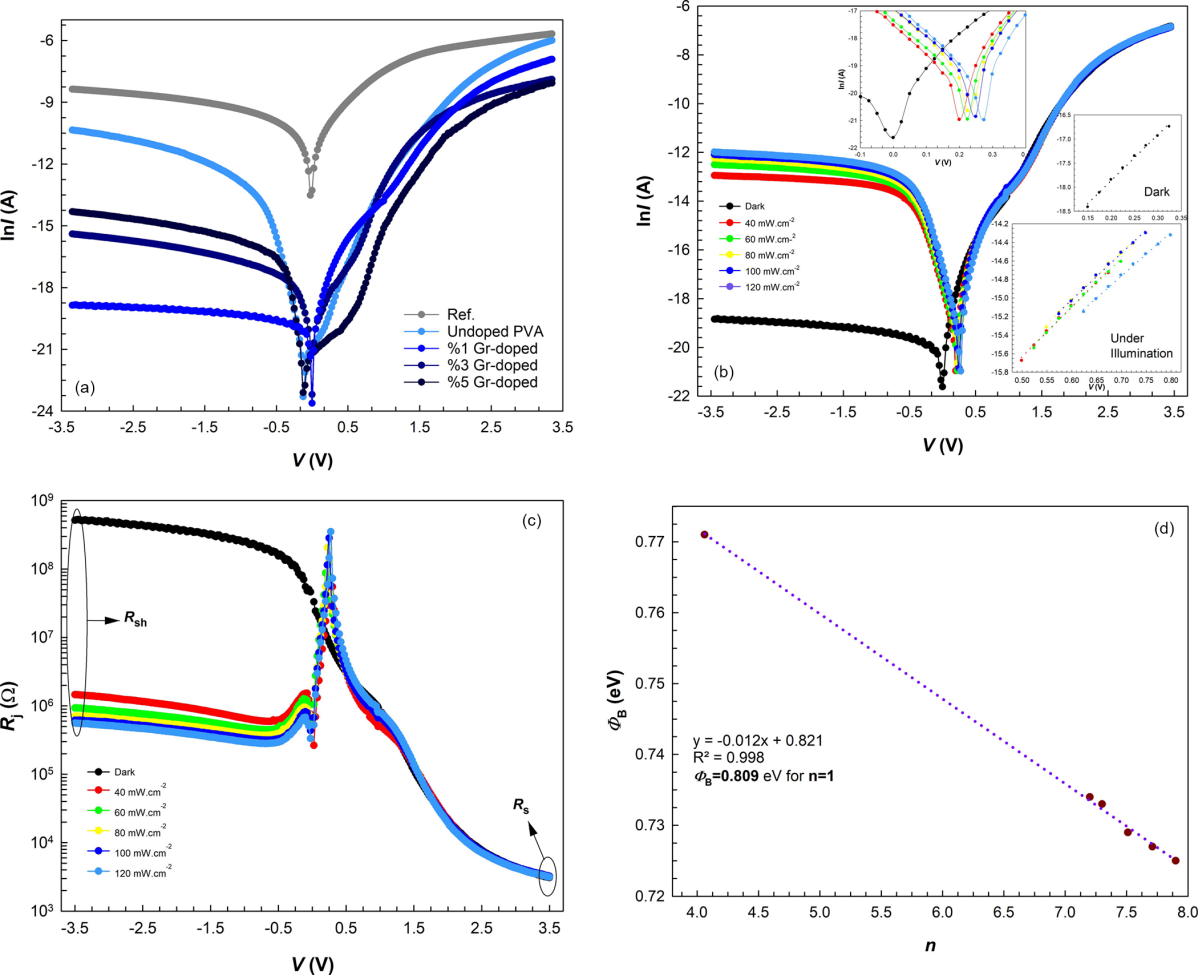
(a) ln *I*–*V* plots of the five specimens in the dark. (b) ln *I*–*V* plots of the preferred diode in the dark and under certain illuminations. (c) *R*_j_–*V* plots of the preferred diode in the dark and under certain illuminations. (d) Plot of the relationship between ϕ_B_ and *n* of the preferred diode obtained from the TE theory.

The basic ln *I*–*V* characteristic plots obtained in the dark measurement of the five different specimens produced are shown in Figure 4a. The main electrical parameters and photodiode characteristics were examined through the data of the specimen with the best diode behavior with the highest rectification ratio (RR) and optimal forward bias linear region (%1 Gr-doped PVA).

First, we applied the standard thermionic emission (TE) theory to establish the forward bias ln *I*–*V* parameters of the diode while in the dark and exposed to certain levels of illumination. Regarding this theory, the *I*–*V* relation for *V*–*IR*_s_ larger than 3*kT*/*q* is given by,^31^
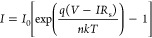
1awhere *V*–*IR*_s_ = *V*_d_ stands for the diffusion potential, including the potential across the series resistance (*IR*_s_). The saturation current (*I*_0_) can be written as the logarithm of both sides of eq 1a.
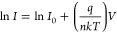
1b

In eq 1b, for the linear region of the ln *I*–*V* plot, *IR*_s_ is not taken into account, and the (−1) next to the exponential term can be neglected because it is minuscule compared to the exponential term. From this equation, *I*_0_ is obtained by the linear portion of the ln *I*–*V* plot with the intersection of the fit line at *V* = 0 V. The expression of *I*_0_ as a function of the barrier height (ϕ_B_) is defined as follows,
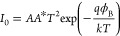
2awhere *k* is the Boltzmann constant, *T* is the room temperature in K, *q* is the electronic charge, *A* is the area of the Al rectifier contacts (∼7.85 × 10^–3^ cm^2^), and A* is the Richardson constant for p-type Si.^31^ The variation of junction ϕ_B_ can be obtained with the *I*_0_ values employing the same equation (eq 2a). The values of *n* were obtained from the slope of the ln *I*–*V* plots for the dark and each illumination, according to the following relation,
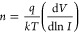
2b

Figure 4b shows the ln *I*–*V* plots of the preferred diode from which the aforementioned basic parameters are obtained. The fact that the diode exhibits remarkable rectification in the dark (RR ∼ 1.67 × 10^5^ at ±3.5 V) and then generates high photocurrent values when illuminated indicates that it has good photodiode characteristics.^32^ As can be understood from this situation, the diode interlayer structure with the best insulator layer among the specimens also exhibited an excellent active layer property, thanks to the high e^–^–h^+^ pair density created by the effect of light falling on it.^33−35^

Determining the shunt (*R*_sh_) and series (*R*_s_) resistances is crucial since these variables significantly impact the electrical characteristics of the system. To calculate the values of *R*_sh_ and *R*_s_, Ohm’s law was basically utilized, and Figure 4c demonstrates the fluctuations in the junction resistance (*R*_j_) obtained from all measurements over the executed voltage range. It can be seen that *R*_sh_ decreases significantly with increasing photocurrent, depending on the illumination intensity. On the other hand, *R*_s_ is almost not affected by illumination intensities. *R*_s_ usually makes its presence felt in the structure depending on the semiconductor bulk resistance, contacts, and/or measurement systems.^36^ These basic parameters obtained are listed in Table 2. It is observed that the *n* values obtained from both techniques increase with increasing illumination intensities, while the ϕ_B_ decreases. As more light-induced electron–hole pairs are created at the junction region with increasing illumination intensity, a great number of electrons cross the barrier and migrate to the metal, causing the *I*_0_ current to increase, ϕ_B_ to decrease, and *n* to increase. The RR value was altered about 1000 times (RR_Dark_/RR_120 mW/cm_^–2^), mainly due to the significant change in the *R*_sh_.

**Table 2:** Basic Electronic Parameters of the Diode Obtained by TE Theory

*P* (mW cm^–2^)	TE					
	*n*	ϕ_B_ (eV)	*I*_0_ (nA)	*R*_s_ (kΩ)	*R*_sh_ (MΩ)	RR
0	4.06	0.771	2.62	3.08	516.44	1.67 × 10^5^
40	7.20	0.734	11.10	3.14	1.46	4.65 × 10^2^
60	7.30	0.733	11.56	3.17	0.93	2.95 × 10^2^
80	7.51	0.729	13.63	3.13	0.71	2.28 × 10^2^
100	7.71	0.727	14.82	3.23	0.62	1.91 × 10^2^
120	7.90	0.725	15.71	3.22	0.54	1.67 × 10^2^

When the variation between ϕ_*B*_ and *n* obtained from the TE theory is plotted (in Figure 4d), it can be seen that there is a fairly linear relation.^37^ Based on this relation, the mean ϕ_B_ for *n* = 1 was found to be about 0.809 eV. The fact that the obtained *n* values are larger than the ideal case (*n* = 1) indicates that not only the TE theory but also different conduction mechanisms, such as generation-recombination (GR) processes and tunneling effect (field emission: FE) and thermionic field emission (TFE), are effective in current-conduction mechanisms (CCMs).^38−41^ However, inhomogeneities in the barrier height also cause the *n* values to deviate from the ideal case. No matter how much attention is paid to the surface cleaning or the interlayer preparation processes during the fabrication stages, the impurities formed in the structure lead to inhomogeneities in the ϕ_B_ and surface/interface states localized in the forbidden bandgap. These cases also increase the *n* values and decrease the ϕ_B_ values.^42−45^ The barrier’s indented structure causes the light-excited charge carriers to flow through its lower energy regions, thereby upping the current and leading to high ideality factor values.^46−48^ In fact, sometimes, the forward bias ln *I*–*V* curves exhibit two or three distinct linear regions due to this inhomogeneity.^49,50^

Furthermore, the basic parameters obtained from the TE theory up to this part were compared to the Cheung and Norde methods. These methods were employed to examine how *n* and ϕ_B_ variations compared to TE theory and determine the *R*_s_ values that are effective at higher forward bias, i.e., nonlinear voltage region of the ln *I*–*V* plots with different approaches. According to the Cheung method, the relations from which *n*, ϕ_B_, and *R*_s_ are obtained are as follows:^51^
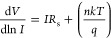
3a

and
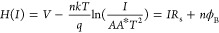
3b

**Figure 5:**
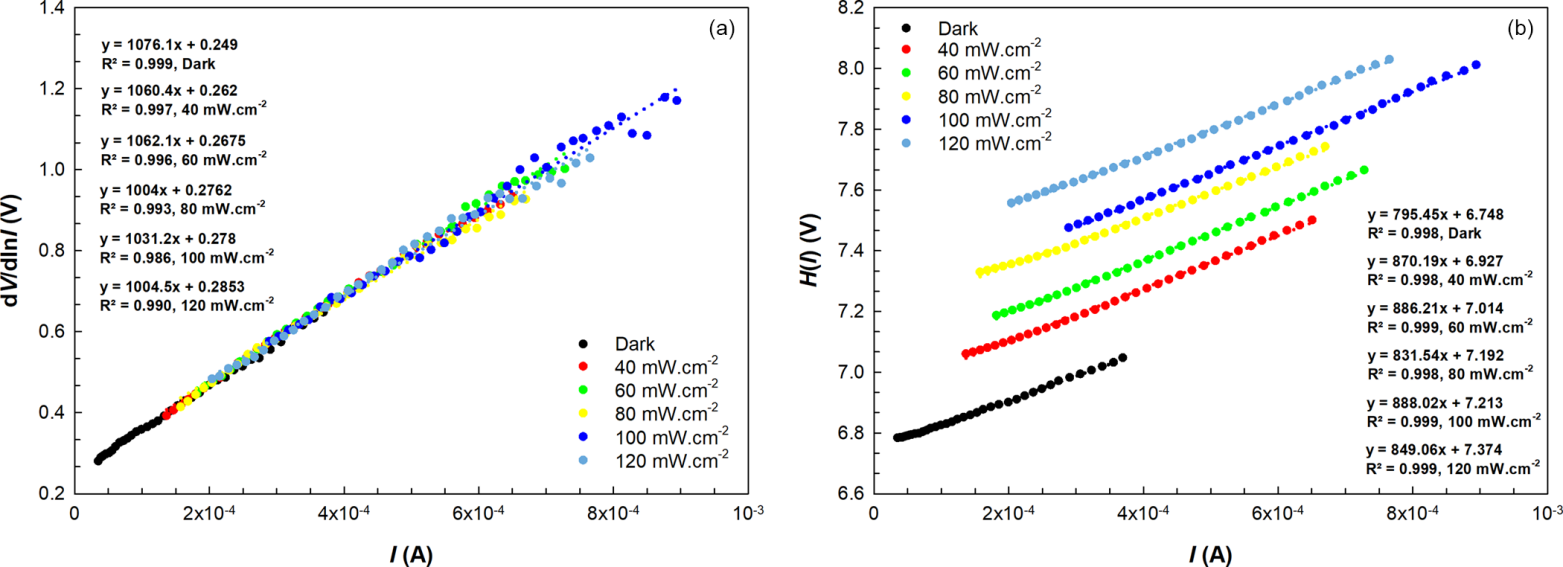
(a) d*V*/d ln*I* vs *I* and (b) *H*(*I*) vs *I* plots obtained from Cheung functions in the dark and under certain illuminations.

The parameters obtained from both methods and their variation as a function of illumination intensities are listed in Table 3. Figure 5a,b shows the d*V*/d ln *I* and *H*(*I*) vs *I* plots in the dark and under certain illumination intensities. The *n* and *R*_s_ values were obtained from the slope and intercept of the d*V*/d ln*I* vs *I* plots. At the same time, the ϕ_B_ and *R*_s_ values were obtained from the slope and intercept of the *H*(*I*) vs *I* plots. The basic parameters obtained by this method are shown in Table 3, and the attitudes of the parameters toward the illumination intensities are similar to those in the TE theory.

**Table 3:** Basic Electronic Parameters of the Diode Obtained by Employing the Cheung and Norde Functions

*P* (mW cm^–2^)	Cheung I, d*V/*d ln*I*		Cheung II, *H*(*I*)		Norde, *F*(*V*)	
	*n*	*R*_s_ (kΩ)	ϕ_B_ (eV)	*R*_s_ (kΩ)	ϕ_B_ (eV)	*R*_s_ (kΩ)
0	9.64	1.08	0.700	0.80	0.953	2.55
40	10.13	1.06	0.684	0.87	0.840	1.02
60	10.34	1.06	0.678	0.89	0.839	0.86
80	10.67	1.00	0.674	0.83	0.831	0.83
100	10.74	1.03	0.672	0.89	0.825	0.81
120	11.02	1.00	0.669	0.85	0.815	0.93

In addition, the Norde method was employed to interpret the effect of the illumination intensities on ϕ_B_ and *R*_s_ that significantly affect the forward ln*I*–*V* characteristic. For this method, the relationship can be given as follows,^52^
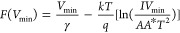
4where γ is an integer greater than the value of *n* obtained.^53^ The *R*_*s*_ and ϕ_*B*_ are described in the following way,
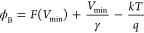
5a
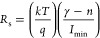
5b

**Figure 6:**
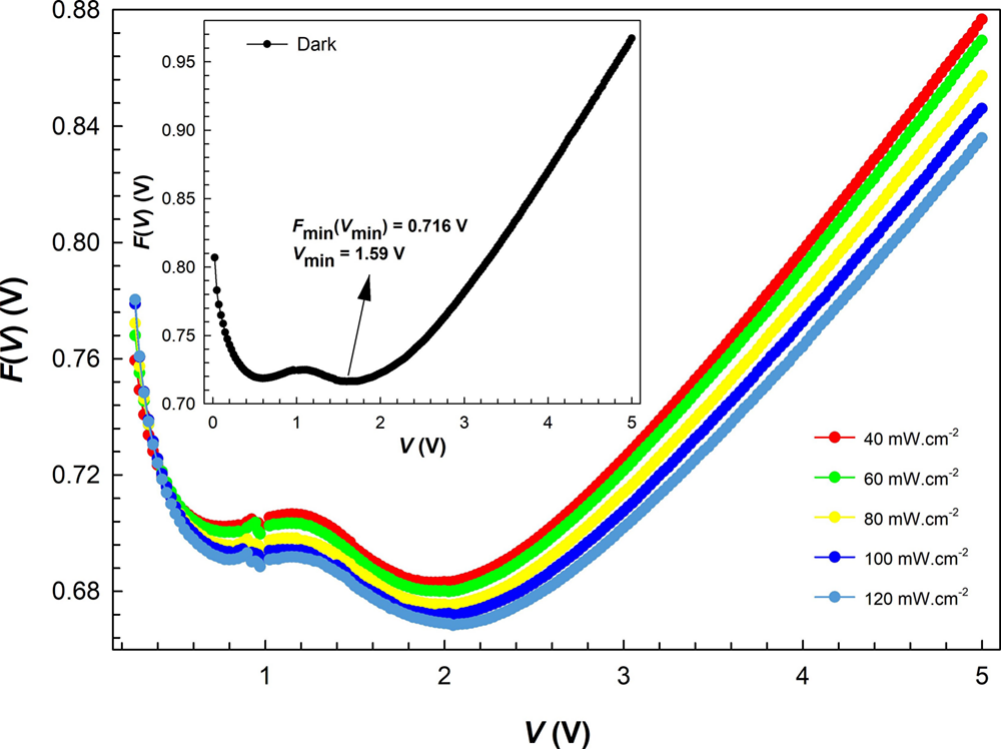
Plots obtained from the Norde function in the dark and under certain illuminations.

The minimum values of the *F*(*V*)–*V* plots are referred to as *F*(*V*_min_), with Imin being the corresponding current to the minimum voltage value (*V*_min_). These values were established at the point where *F*(*V*)–*V* was at its lowest on the curve, as illustrated in Figure 6. Consequently, using the above equations results in values for *n* and *R*_s_.

The tables show that the values of *n* and ϕ_B_ obtained by all three methods change in the same parallel way as a function of the illumination intensity, i.e., the values of *n* increase, while the values of ϕ_B_ decrease. It has been observed that the values of *R*_s_ obtained by the TE theorem and the Cheung method almost do not change with the illumination intensities. However, there is a noticeable decrease in the values obtained by the Norde method, especially under illumination. The difference between the values obtained by the three methods is due to the series resistance, the voltage drop across the interlayer, and the voltage range dependence of the electrical parameters (linear or nonlinear region of ln*I*–*V*).^54−56^

Before moving on to the photoresponse characteristics of the structure, it would be appropriate to address the interface and surface states (*N*_ss_) and their distributions, which significantly affect the CCM, at the end of this section. The variation of these states, which have different sources in the structure interlayer,^57^ depending on the illumination intensities and the difference in energy levels, was examined with the method brought out by Card and Rhoderick.^58^ Accordingly, the necessary relation to obtaining *N*_ss_ is given below,
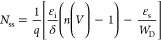
6

The energy levels of the interface states (*E*_ss_) are calculated with respect to the edge of the valence band (*E*_v_) for p-type Si and are given by,
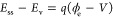
7where ϕ_e_ is the effective barrier height, which is provided by,
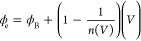
8

**Figure 7:**
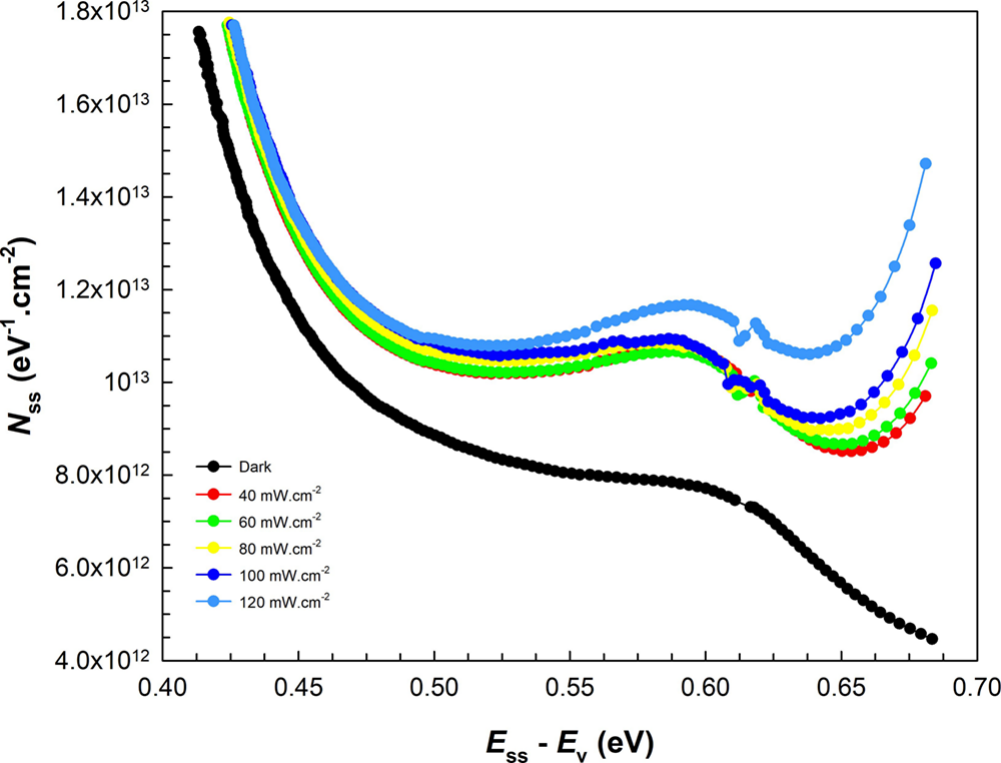
*N*_ss_ vs *E*_ss_–*E*_v_ plots of the diode in the dark and under certain illuminations.

The plots of *N*_ss_ vs *E*_ss_–*E*_v_ obtained from the above equations are given in Figure 7. It can be clearly seen that these states (*N*_ss_) decrease almost exponentially with increasing energy differences (*E*_ss_–*E*_v_) in the dark, while they begin to increase in the deep trap levels for each illumination intensity. It can be said that the illuminations that create charge pairs at the interface also cause an increase in the interface/surface states at deep trap levels.^59−65^

### 3.2 Photodetector Characteristics

**Figure 8:**
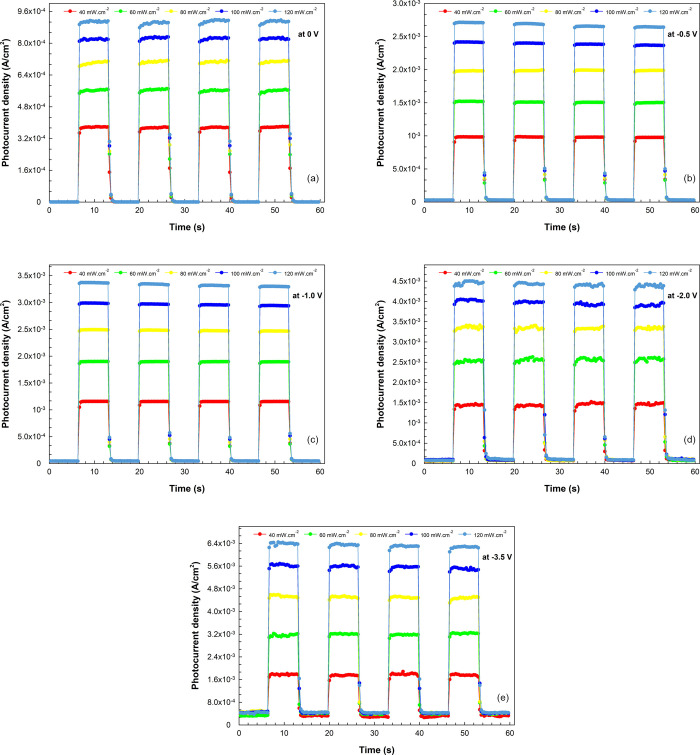
(a–e) Photocurrent vs time plots of the diode under certain illuminations and voltages.

**Figure 9:**
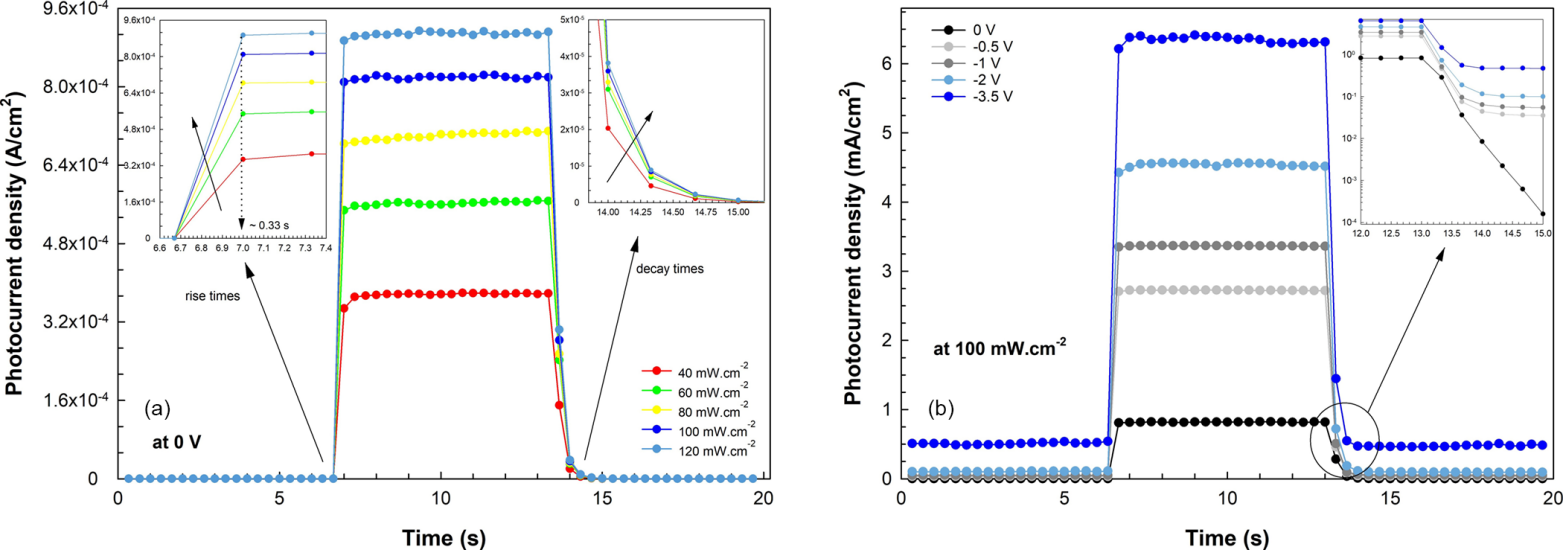
(a) Transient photocurrent density and (inset) the rise and decay times of the diode under certain illuminations. (b) Photocurrent density and the (inset) decay times of the diode at certain biases under 100 mW cm^–2^.

In this section, the sensitivity (*S*), responsivity (*R*), and specific detectivity (D*) of the structure under certain illumination intensities were examined. First, the transit photocurrent characteristic at zero bias and then the photocurrent alterations at certain reverse biases were examined, depending on the increasing illumination intensities for one-minute durations. Figure 8a–e demonstrates that the photocurrent density exhibits significant variation with changes in illumination intensity. For instance, if we concentrate on the intensity of 100 mW cm^–2^ and implement a single-doped interlayer within the structure, the value of the transient photocurrent density (refer to Figure 8a) is adequate for comparison against analogous research in the literature.^10,66,67^ Again, for the transient photocurrent values in Figure 9a, the rise times are approximately 0.33 s under all illumination intensities (independent of the illumination).

In contrast, the decay times decrease with increasing illumination intensities. This variation in the decay times can be attributed to the redistribution of the intrinsic electric field due to the trapped charges at the interface.^68,69^ Furthermore, it is evident from Figure 7 that the illumination intensity can influence the variation of shallow and/or deep trap levels. With higher levels of illumination, greater intensities of trap levels may quicken the processes of charge separation and recombination at the interface through a process known as the GR process.^70−72^ Thus, the photosensitive properties of the structure can change depending on the density of interface states/trap levels and the trapping/detrapping lifetimes.^73,74^

Considering the generation of steady-state conditions, it can be said that slightly wavy transient photocurrents occur due to the interface states/trap level effect of the charge carriers formed under illumination when no electric field is applied to the structure (at zero bias). However, it can be addressed that the relatively weak electric fields formed at lower bias voltages (at −0.5 and 1 V) make the photocurrents more stable, with some drift currents where scattering is not dominant. As expected, the photocurrent density gradually increases at higher reverse biases (at −2.0 and −3.5 V). However, deviations from linearity occur at steady-state conditions due to scattering effects in the charge drift and the increase in dark current density.^69,75^ Meanwhile, the decay dynamics become faster as the internal electric field gradually increases from 0 to −3.5 V, as shown in Figure 9b.

**Figure 10:**
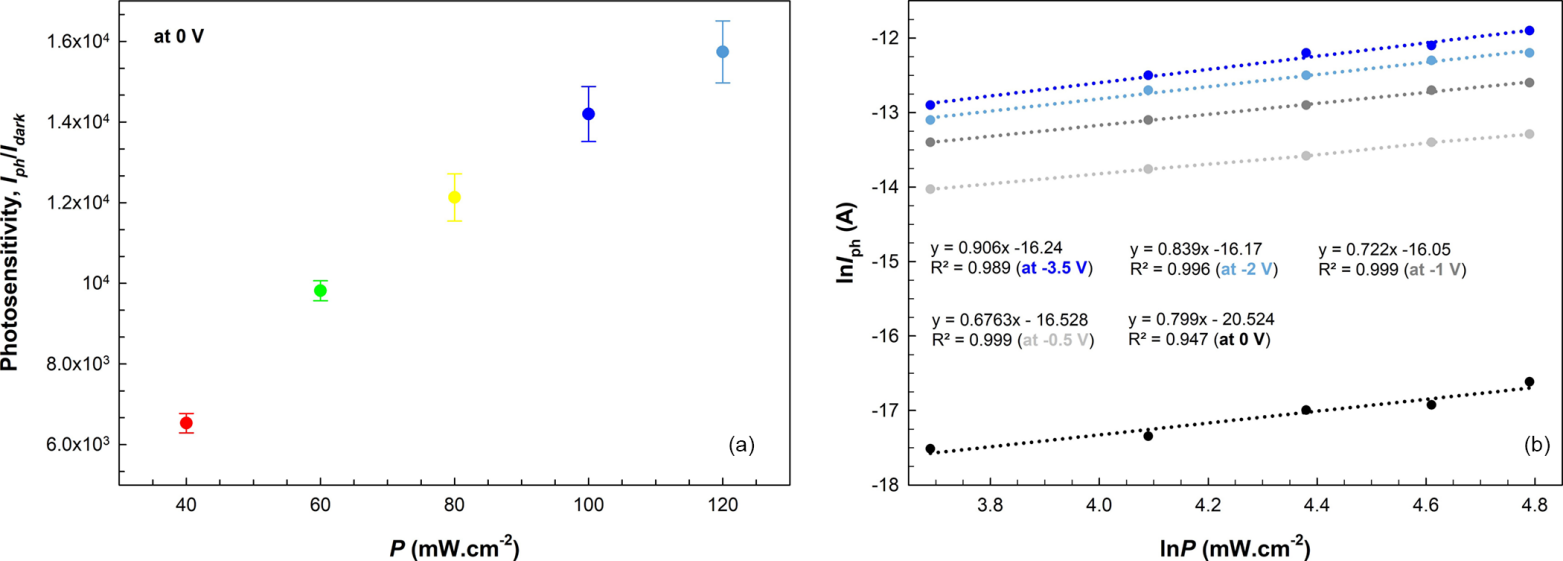
(a) Photosensitivity values of the diode under certain illuminations at zero bias. (b) ln *I*_ph_ vs ln *P* plots of the diode at zero and reverse biases under certain illuminations.

Photosensitivity, or sensitivity (*S*) for short, of optoelectronic devices is defined as the ratio of photocurrent to dark current (*S* = *I*_ph_/*I*_dark_).^76^ The *S* plots of the diode for certain illumination intensities are shown in Figure 10a at zero bias. As a result of the high increase in transient *I*_ph_ values and low dark current, it is an expected result that the *S* values reach high values with the illumination intensities. It is affirmed that the diode exhibits high photosensitivity.^77,78^ In addition, the power law expresses the correlation between photocurrent (*I*_ph_) and illumination intensity (*P*), which is given by the equation,^79^
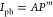
9where *A* is a constant. The ln *I*_ph_ vs ln *P* plots are shown in Figure 10b to determine *m* values at different negative voltages. The slopes of the plots, which show good linear behavior, and thus the *m* values obtained, are also inset. The alteration in these values from 0.906 to 0.799 between −3.50 and 0 V indicates that the existence of minority-carrier traps/states and responsivity tends to decrease under high illumination levels. In other words, the diode demonstrates photoconductive behavior, which is also evident in the photosensitivity values.^78,80^

The responsivity of a PD is defined as the ratio of the photocurrent density (*J*_ph_) to the incident illumination intensity (*P*) and is given as follows,^81^
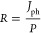
10

**Figure 11:**
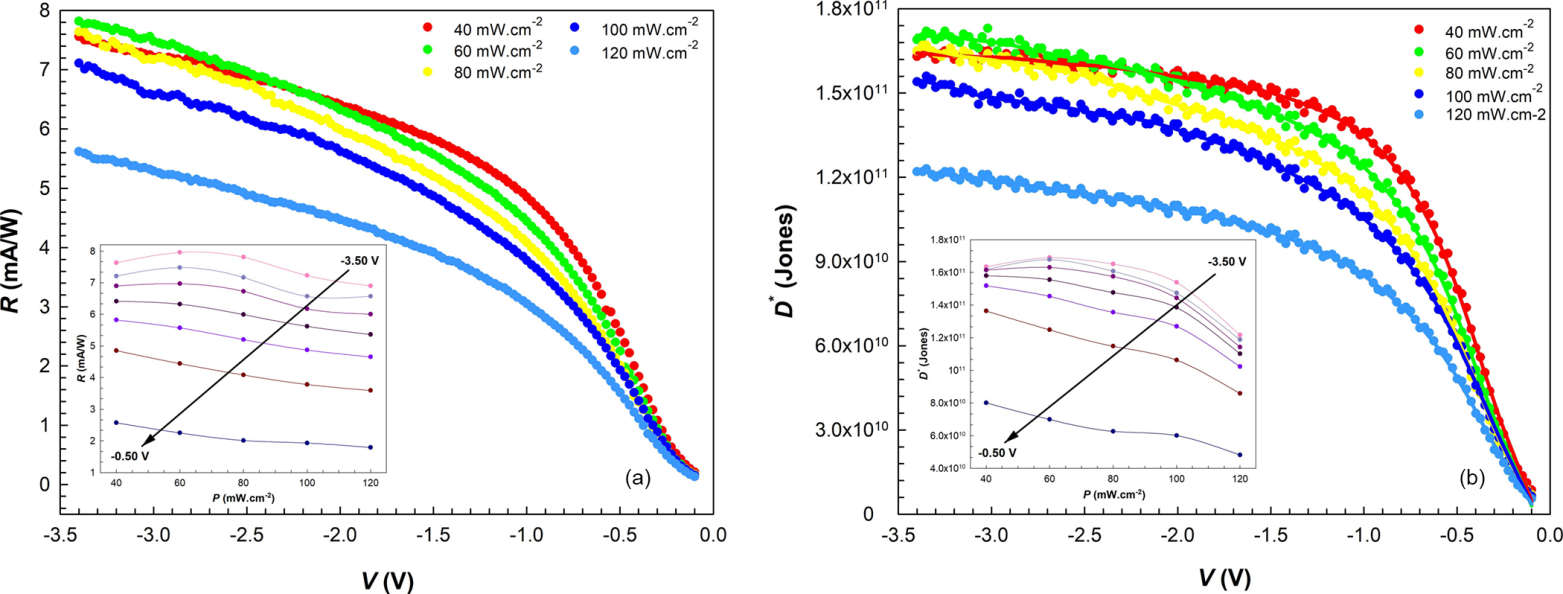
(a) *R* vs *V* plots of the diode under certain illuminations and (inset) the *R* vs *P* plots at certain biases. (b) D* vs *V* plots of the diode under certain illuminations and (inset) the D* vs *P* plots at certain biases.

Figure 11a shows the altering of the *R* depending on *P* in the reverse bias. The *R* values decrease with increasing illumination intensity due to the saturation of the levels that trap electrons at high illumination intensities.^82,83^ It should be noted that the *R* values also increase at all illumination intensities as the internal electric field strength increases (from 0.0 to −3.5 V).^84,85^

The specific detectivity (D*) is one of the essential parameters for optoelectronic devices, which describes the smallest detectable signal and can be estimated by the equation as follows,
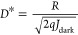
11where *J*_dark_ is the current density measured in the dark, and D* is in the Jones unit (Jones = cm Hz^0.5^/W). At this point, we can focus on another reason for the diode we chose among the specimens. Since the selected diode has much less leakage current in the dark, both *S* and D* values are quite satisfactory.^86−88^ Still, it is seen that it depends on the illumination intensities proportionally with *R* values (see Figure 11b).^89^ Furthermore, the D* value of the diode was found to be 5.53 × 10^9^ Jones under 120 mW cm^–2^ at zero bias voltage (self-powered mode), which is the situation where no voltage is applied to the structure and the charge carriers create photocurrent only with the diffusion effect. Table 4 presents a comparison of photodetector parameters for similar interlayered structures based on illumination intensities.

**Table 4:** Some Photodetector Parameters from Similar Studies

sample	illumination (mW cm^–2^)	bias (V)	sensitivity, *I*_on_*/I*_off_	responsitivity (A/W)	detectivity (cm Hz^0.5^/W)	rise time (ms)	ref.
p-Si/n-SiC	100	0	∼40	5	7.6 × 10^11^		(90)
*Epilobium angustifolium*/n-Si	10	0	1.1 × 10^3^	0.03	5.65 × 10^11^		(91)
RuO_2_:PVC/n-Si	100	–0.2	∼42	0.3			(92)
graphene/planar-Si	5–30 (×10^–3^) @890 nm	0	10^7^	0.73	5.77 × 10^13^	0.32	(93)
nanographene/planar-Si	1 × 10^–2^	0		0.1–0.3			(94)
graphene/AlN/n-Si	12.2	–10	∼4 × 10^4^	1.03	2.94 × 10^7^	1.9	(95)
	@365 nm						
	@850 nm			3.96	1.13 × 10^8^		
graphene/p-Si	1 × 10^–2^ @460 nm	5		5.5	2.35 × 10^10^		(96)
graphene/p-Si		1		0.13	1.2 × 10^9^		(97)
	@633 nm						
graphene/h-BN/n-Si	6			∼0.1	∼10^13^	0.91	(98)
	@725 nm	0.03					
PBT7/graphene/ODTS/SiO_2_/p^+^-Si	10^–4^	0		∼10^5^		7.8	(99)
P3HT–graphene/Si		–0.1		0.78	2.6 × 10^10^		(100)
	@725 nm						
SLG-CNTF/SiO_2_/Si	166	–3	∼240 @0 V	209	3.87 × 10^10^	68 × 10^–3^	(101)
	@980 nm						
GNWs/DLC/n-Si	0.5	1		2400	1.07 × 10^11^	20 × 10^–3^	(102)
	@532 nm						
graphene/spiro-OMeTAD/n-Si	0.145	0	∼10^7^	0.355	8.7 × 10^10^	5.1 × 10^–3^	(103)
	@532 nm						
polymer/graphene/n-Si	2.8 × 10^–6^	5		1.78 × 10^5^			(24)
graphene:PVA/p-Si	100	0	1.4 × 10^4^	5.7 × 10^–5^	5.35 × 10^9^	∼33	this work

## 4 Conclusions

This paper outlines the characteristics of the photodiode and photodetector for a Schottky structure featuring a Gr-doped PVA interlayer. The *I–V* measurements were conducted in both dark and illuminated conditions while varying the illumination intensity from 40 to 120 mW cm^–2^ in increments of 20 mW cm^–2^. The obtained results confirm that the diode exhibits satisfactory rectification in the absence of illumination. The fact that the currents accrued in the negative region with the illumination intensities (photocurrents) are about 1000 times higher than the dark currents is an essential indication that the structure exhibits good photodiode behavior. The fundamental electrical parameters, such as *n*, ϕ_B_, and *R*_s_, are evaluated by three methods (TE, Norde, and Cheung). Still, the distributions of *N*_ss_, which markedly affect the electrical dynamics of the structure, were observed as a function of the illumination intensities. In this sense, it has been observed that the structure has critical photodiode characteristics. The detailed analysis of the changes in time–response photocurrents resulting from variations in illumination and voltage and how they are influenced by interface states/trap levels was discussed. Additionally, the study examines the rise-decay behavior of these photocurrents. The results indicate that the photodetector characteristics of the structure are strongly influenced by the additional charge carriers and traps that form under the light. Notably, the responsivity, sensitivity, and detectivity are in considerable values and alterations due to high photocurrents that occur depending on the illumination intensities and voltage. The strong relationship between photocurrents and illumination intensities was also plotted as double logarithmic, and good linearity was observed. The fact that the linear slopes are less than 1 over a wide voltage range indicates that the structure is a proper photoconductor. Another critical situation is the ease and nontoxicity of the preparation and coating of the interlayer, both chemically and physically. The intentionally created structure has potential as a photodetector in optical sensor applications. It is evident from our analysis of these situations.
